# Supplementary material for: Profiling the CFTR Variant Selectivity and Off-Target Interactions of VX-121
Source: bioRxiv. 2026 Jun 8:2026.06.01.729306. Preprint. [Version 2] doi: 10.64898/2026.06.01.729306 (PMC13252174; doi:10.64898/2026.06.01.729306)
Supplement: Supplement 1 [file media-1.pdf]

Supplemental Information for:

Profiling the CFTR Variant Selectivity and Off-Target Interactions of VX-121

Ashish R. Jhangiani,[1‡] John A. Olson III,[2,3‡] Austin Tedman,[1] Catherine Foye,[4,5] JaNise J. Jackson,[4,5] Ashlyn G. Winters,[4,5] Janiyah A. White,[4,5] Mia Perfetti,[2] Grace M. Abell,[2] Crissey D. Cameron,[2] Liudmyla Arifova,[3,6] Brianna Corman,[7] J. Paul Robinson,[7] Charles P. Kuntz,[1] Kaitlyn V. Ledwitch,[2] Jens Meiler,[2,8,9] Kathryn E. Oliver,[4,5*] Lars Plate,[2,10,11*] and Jonathan P. Schlebach[1*]

[1]The James Tarpo Jr. and Margaret Tarpo Department of Chemistry, Purdue University, West Lafayette, Indiana, USA
[2]Department of Chemistry, Vanderbilt University, Nashville, TN, USA
[3]Chemical and Physical Biology Program, Vanderbilt University, Nashville, TN, USA
[4]Department of Pediatrics, Emory University, Atlanta, GA, USA
[5]Center for Cystic Fibrosis and Airways Disease Research, Children's Healthcare of Atlanta and Emory University, Atlanta, GA, USA
[6]Department of Biochemistry, Vanderbilt University, Nashville, TN, USA
[7]Weldon School of Biomedical Engineering, Purdue University, West Lafayette, IN, USA
[8]Center for Structural Biology, Vanderbilt University, Nashville, TN, USA
[9]Institute for Drug Development, Leipzig University, Leipzig, SAC, Germany
[10]Department of Biological Sciences, Vanderbilt University, Nashville, TN, USA
[11]Department of Pathology, Microbiology and Immunology, Vanderbilt University Medical Center, Nashville, TN USA

‡ Authors contributed equally

*Corresponding Authors: jschleba (at) purdue.edu, lars.plate (at) vanderbilt.edu, kolive3 (at) emory.edu,

Contents:

-Figure S1
-Figure S2
-Table S1

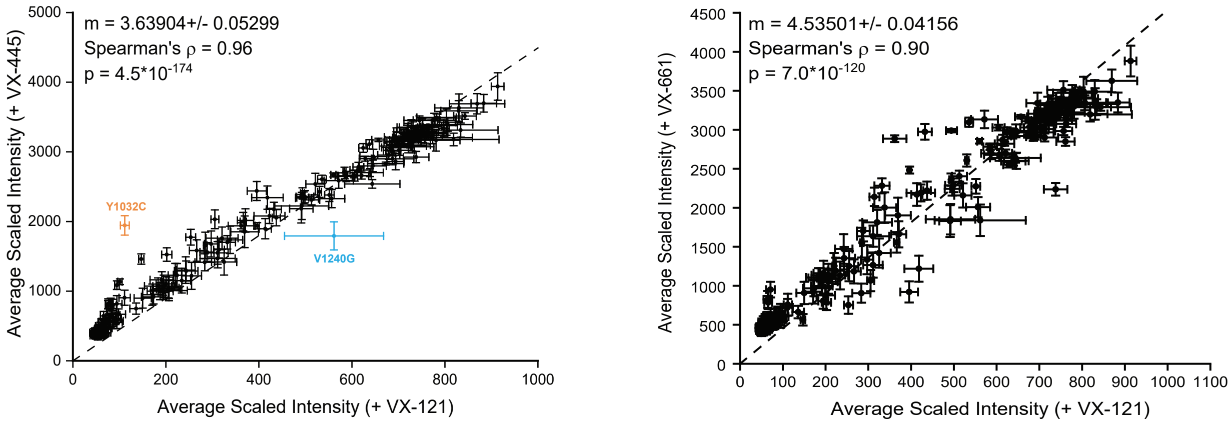

Figure S1. Comparison of Surface Immunostaining Intensities of CF Variants in the Presence of VX-121, VX-445, and VX-661. The surface immunostaining intensities of 232 CF variants were determined in the presence of 3 μM VX-121 by deep mutational scanning. Intensity values in the presence of VX-121 are plotted against the corresponding intensities in the presence of A) 3 μM VX-445 or B) 3 μM VX-661 Measurements represent the average of 3 biological replicates. The error bars represent Standard Error. A linear fit line (black dashes) is shown for reference and the fitted values of the slope (*m*) is shown along with the and Spearman's Correlation coefficient (ρ), for reference.

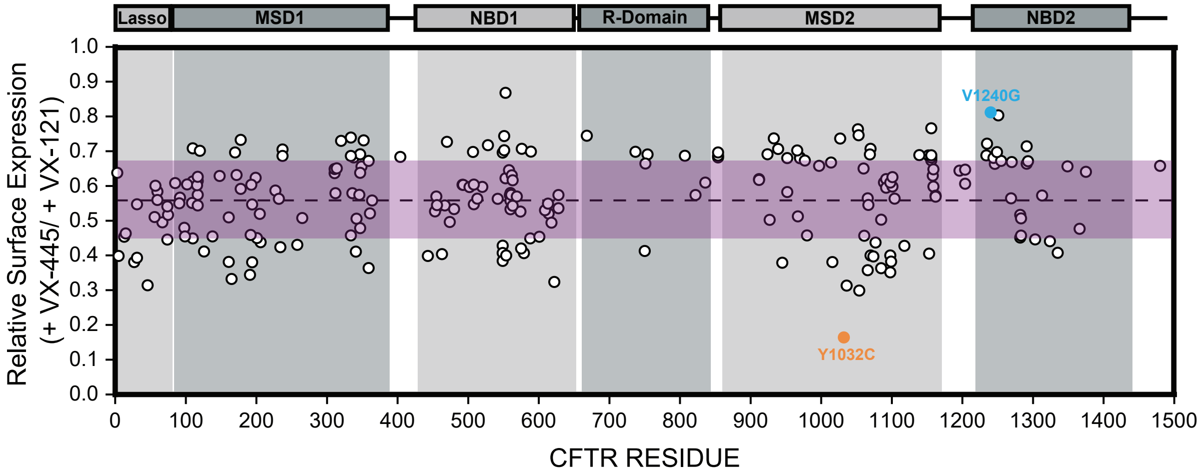

Figure S2. Alternative Scaling for CF Variants VX-121/ VX-445 Response Ratio. CFTR surface immunostaining intensities for each variant in the presence vehicle were used to scale the corresponding intensity values in the presence of 3 μM VX-121 or 3 μM VX-445. The ratio of the VX-445-treated intensity to the corresponding VX-121-treated intensity for each variant is plotted against the position of the mutated residues within the CFTR sequence. The boundaries of the lasso motif, membrane spanning domain 1 (MSD1), nucleotide binding domain 1 (NBD1), the R-domain, membrane spanning domain 2 (MSD2), and nucleotide binding domain 2 (NBD2) are highlighted in gray for reference, and the purple stripe reflects values within one standard deviation of the mean. Trends are similar to the reciprocol VX-121/ VX-445 ratios that are plotted in Figure 1E.

Table S1. Deep Mutational Scanning Measurements of CF Variant Immunostaining Intensities.

CFTR Sub-Domain	Variant	Amino Acid Substitution	Vehicle Control -Average Scaled Intensity (For VX-121)	VX-121 Average Scaled Intensity	Up-Scaled VX-121 Average Scaled Intensity	Vehicle Control - Average Scaled Intensity (For VX-445)	VX-445 Average Scaled Intensity	Vehicle Control - Average Scaled Intensity (For VX-121+VX-661)	VX-121+VX-661 Average Scaled Intensity
Lasso	R3W	7A>T	315.2	646.2	1871.0	912.6	2934.1	741.0	3352.7
Lasso	P5L	14C>T	105.6	395.4	971.5	259.6	2438.3	123.7	1959.3
Lasso	S13F	38C>T	56.1	77.5	279.6	202.4	616.2	63.1	579.5
Lasso	L15P	44T>C	60.9	71.1	248.0	212.3	535.3	79.8	507.8
Lasso	G27R	79G>A	55.4	66.2	245.2	205.3	644.5	64.9	517.5
Lasso	R31C	91C>T	215.2	492.2	1216.1	531.7	2223.6	448.6	2135.9
Lasso	R31L	92G>T	159.8	418.3	920.7	351.8	2342.9	179.1	2037.7
Lasso	A46D	137C>A	70.0	147.1	458.7	218.2	1460.9	67.7	1017.9
Lasso	E56K	166G>A	59.3	64.9	227.3	207.7	445.6	57.3	821.3
Lasso	W57G	169T>G	44.7	52.8	235.6	199.6	392.3	71.3	303.6
Lasso	E60X	178G>T	48.5	53.7	229.1	206.8	392.5	71.2	363.7
Lasso	E60K	178G>A	47.0	51.4	217.0	198.1	387.9	63.7	466.7
Lasso	P67L	200C>T	63.1	65.8	221.5	212.4	447.5	77.3	733.8
Lasso	R74W	220C>T	214.5	288.3	695.9	517.8	1562.0	385.8	1513.6
Lasso	R74Q	221G>A	288.2	432.2	1175.8	784.1	2174.8	626.3	3242.0
Lasso	R75Q	224G>A	274.2	361.6	1001.1	759.2	1937.8	589.3	2898.0
TMD1	G85E	254G>A	45.3	52.7	233.3	200.2	383.8	62.9	306.7
TMD1	G91R	271G>A	51.1	59.1	224.9	194.5	409.0	71.3	654.5
TMD1	E92K	274G>A	50.3	56.6	228.9	203.7	404.6	65.4	338.2
TMD1	Q98R	293A>G	63.7	71.1	234.9	210.3	490.2	71.9	915.1
TMD1	P99L	296C>T	188.9	283.8	642.7	427.8	1414.8	286.7	1083.5
TMD1	L102R	305T>G	43.2	50.0	230.2	199.1	381.1	57.4	416.5
TMD1	Y109N	325T>A	275.6	396.3	1068.0	742.7	1938.7	695.2	2980.0
TMD1	D110Y	328G>T	217.5	314.4	715.9	495.4	1594.4	370.4	2264.9
TMD1	D110E	330C>A	441.2	756.4	2291.4	1336.6	3237.4	1188.1	3081.6
TMD1	D110H	328G>C	357.3	535.7	1596.4	1064.9	2606.2	868.5	2757.1
TMD1	E116K	346G>A	344.5	494.3	1409.2	982.0	2342.6	829.9	2991.9
TMD1	R117C	349C>T	322.4	513.1	1326.1	833.3	2310.2	679.5	2418.5
TMD1	R117H	350G>A	362.4	560.1	1638.1	1059.8	2668.3	825.4	2722.5
TMD1	R117P	350G>C	356.9	606.2	1662.6	978.8	2655.8	905.9	3200.9
TMD1	R117L	350G>T	285.2	422.7	1130.1	762.4	2079.8	621.4	2570.3
TMD1	A120T	358G>A	431.6	747.8	2255.2	1301.5	3217.0	1116.2	3501.5
TMD1	G126D	377G>A	158.2	242.5	535.9	349.5	1302.7	193.7	1791.6
TMD1	L138ins	413_415dupTAC	53.5	58.8	221.1	201.2	486.5	65.6	565.4
TMD1	I148T	443T>C	368.9	571.6	1625.7	1049.1	2588.0	886.0	2830.0
TMD1	Y161D	481T>G	48.8	53.3	217.6	199.3	427.2	59.2	476.2
TMD1	Y161C	482A>C	60.0	83.0	285.4	206.4	748.6	61.7	907.8
TMD1	L165S	494T>C	56.4	75.8	267.7	199.1	806.1	71.6	505.5
TMD1	R170H	509G>A	401.7	679.3	2055.6	1215.7	2953.4	1077.9	3350.9
TMD1	L172I	514C>A	362.3	637.5	1963.0	1115.7	3109.0	868.3	3209.2
TMD1	G178R	532G>A	375.8	585.0	1570.3	1008.9	2655.4	781.9	2691.3
TMD1	G178E	533G>A	444.6	806.2	2483.0	1369.1	3391.7	1214.5	3472.2

TMD1	F191V	571T>G	89.6	201.9	524.8	233.0	1525.5	93.6	1665.8
TMD1	D192G	575A>G	78.0	253.4	814.4	250.6	1774.5	103.9	1299.2
TMD1	E193K	577G>A	303.8	530.4	1463.0	838.1	2425.6	685.2	3174.1
TMD1	G194R	580G>A	287.6	521.2	1386.5	765.0	2537.4	647.4	2809.7
TMD1	G194V	581G>T	142.0	220.0	469.0	302.6	1233.5	192.9	1826.9
TMD1	H199Y	595C>T	48.5	58.7	243.7	201.4	391.2	74.9	391.1
TMD1	V201M	601G>A	215.3	331.9	765.4	496.5	1701.1	380.2	2362.5
TMD1	P205S	613C>T	50.3	55.6	226.7	205.1	436.6	57.5	388.7
TMD1	L206W	617T>G	54.0	64.9	243.2	202.5	555.6	69.0	561.4
TMD1	L227R	680T>G	47.4	55.7	235.3	200.3	401.2	80.9	411.4
TMD1	V232D	695T>A	51.3	63.6	250.7	202.2	445.8	64.9	471.0
TMD1	A234D	701C>A	200.5	311.0	656.2	423.1	1549.3	325.0	1802.9
TMD1	Q237E	709C>G	428.4	762.7	2312.7	1299.0	3282.1	1118.6	3353.9
TMD1	Q237H	711G>C	441.2	788.1	2391.9	1339.2	3486.7	1155.7	3595.3
TMD1	R258G	772A>G	100.8	136.0	322.1	238.7	749.1	91.9	889.9
TMD1	M265R	794T>G	72.6	94.5	289.8	222.7	571.2	61.6	602.8
TMD1	F311L	933C>G	411.2	686.0	1999.7	1198.8	3140.8	940.6	2962.5
TMD1	F311L	933C>A	413.8	712.4	2054.9	1193.7	3168.7	961.4	3296.1
TMD1	F312DEL	935_937del	267.2	556.5	1347.5	647.1	2328.7	574.0	2787.1
TMD1	G314E	941G>A	325.3	639.1	1757.2	894.4	2702.6	761.0	3062.9
TMD1	L320V	958T>G	450.7	803.6	2498.1	1401.0	3425.2	1190.4	3540.4
TMD1	R334W	1000C>T	461.9	797.6	2467.8	1429.3	3338.9	1184.9	3227.9
TMD1	R334L	1001G>T	73.9	92.8	275.3	219.2	602.2	89.3	510.2
TMD1	R334Q	1001G>A	441.0	756.9	2248.4	1310.1	3276.8	1095.5	3304.7
TMD1	I336K	1007T>A	54.1	72.3	271.3	202.9	468.7	70.3	562.9
TMD1	T338I	1013C>T	407.9	721.3	2111.6	1194.0	3098.1	985.6	2767.5
TMD1	S341P	1021T>C	166.9	243.5	529.8	363.1	1289.8	230.2	1586.8
TMD1	R342W	1054C>T	214.1	311.2	742.9	511.1	1469.8	355.1	1974.5
TMD1	L346P	1037T>C	43.5	51.4	221.9	188.0	386.6	64.1	559.2
TMD1	R347H	1040G>A	484.7	883.6	2553.5	1400.7	3697.1	1218.9	3036.6
TMD1	R347P	1040G>C	90.5	200.5	528.4	238.5	1104.3	134.2	1249.2
TMD1	R347P	1040G>T	339.5	738.1	1865.3	858.1	2925.4	849.1	3110.9
TMD1	A349V	1046C>T	358.4	604.7	1746.9	1035.5	2663.9	844.6	2642.9
TMD1	R352Q	1055G>A	467.6	843.1	2568.3	1424.3	3514.9	1212.0	3118.8
TMD1	Q359K/T360K	1075-1079C>A	151.5	266.3	575.6	327.4	1582.8	200.5	1564.8
TMD1	Q359R	1076A>G	403.2	689.0	2023.5	1184.1	3011.4	1069.9	3464.4
TMD1	W361R	1081T>C	52.0	59.6	222.6	194.2	427.5	68.6	418.0
TMD1	S364P	1090T>C	51.8	58.6	236.1	208.8	423.3	53.9	397.7
XXX	G404R	1210G>C	416.1	695.8	2194.1	1312.0	3212.0	1058.9	3405.7
NBD1	D443Y	1327G>T	165.5	195.3	426.6	361.5	1070.9	236.3	1450.2
NBD1	L453S	1358T>C	54.5	58.7	262.9	243.8	499.1	63.7	424.6
NBD1	A455E	1364C>A	51.4	59.4	227.8	197.3	400.0	71.4	343.4
NBD1	V456A	1367T>C	48.5	52.6	214.5	197.8	398.1	103.6	424.0
NBD1	V456F	1366G>T	48.8	53.4	222.3	203.4	408.0	73.0	386.3
NBD1	G463D	1388G>T	54.2	79.8	294.9	200.2	731.1	58.2	585.6
NBD1	L467P	1400T>C	47.2	49.9	199.6	188.9	367.3	63.0	416.2

NBD1	M470V	1408A>G	417.0	645.1	2033.6	1314.6	2798.3	1038.3	2354.1
NBD1	E474K	1420G>A	48.8	55.9	224.3	195.8	452.4	70.7	437.5
NBD1	G480S	1438G>T	53.5	60.5	233.2	206.2	437.5	69.2	420.0
NBD1	S492F	1475C>T	50.3	58.1	225.8	195.5	374.3	73.5	345.7
NBD1	Q493X	1477C>T	44.3	51.8	231.5	198.0	384.3	64.3	360.0
NBD1	I502T	1505T>C	50.1	56.3	224.0	199.5	375.4	67.4	329.6
NBD1	I507del	1519_1521delATC	48.3	61.9	254.7	198.8	365.2	64.0	379.4
NBD1	F508del	1521_1523delCTT	50.3	57.9	224.5	195.1	410.3	73.1	375.1
NBD1	F508C	1523T>G	343.5	624.3	1844.4	1014.7	3055.5	876.2	3414.7
NBD1	D513G	1538A>G	49.2	56.2	227.4	198.7	403.7	62.8	400.3
NBD1	V520F	1558G>T	46.6	52.4	226.5	201.4	379.6	68.3	364.7
NBD1	E528E	1584G>A	436.4	761.3	2352.7	1348.7	3280.6	1095.0	3400.6
NBD1	G542X	1624G>T	41.5	45.9	226.7	205.2	402.9	64.8	407.7
NBD1	S549R	1645A>C	188.7	205.4	439.3	403.7	1027.0	287.9	1177.7
NBD1	S549N	1646G>A	442.8	741.6	2244.1	1340.0	3226.6	1114.2	2879.0
NBD1	S549R	1647T>A	174.2	183.3	436.8	414.9	1072.9	267.6	1136.7
NBD1	S549R	1647T>G	187.6	194.2	398.1	384.7	1036.1	307.5	1027.9
NBD1	G551S	1651G>A	470.1	869.4	2741.6	1482.6	3690.0	1245.6	3317.3
NBD1	G551D	1652G>A	470.6	829.5	2519.1	1429.1	3587.9	1149.3	3232.5
NBD1	R553X	1657C>T	44.3	54.7	250.3	203.1	402.5	70.3	357.6
NBD1	R553N	1658G>A	395.9	818.7	2757.7	1333.6	3178.8	1173.8	3457.7
NBD1	A554E	(1661C>A)	155.3	173.3	367.2	329.2	918.2	219.9	1249.9
NBD1	L558S	1673T>C	50.4	61.6	235.4	192.7	364.8	69.0	328.2
NBD1	A559T	1675G>A	52.5	60.0	228.1	199.5	397.9	83.6	326.8
NBD1	R560K	1679G>A	44.7	48.7	213.4	195.8	368.4	66.5	342.2
NBD1	R560T	1679G>C	50.9	55.4	220.2	202.2	395.6	88.6	371.9
NBD1	R560S	1680A>C	49.3	52.4	203.7	191.5	382.4	65.0	460.6
NBD1	A561E	1682C>A	47.0	52.3	215.9	193.9	376.0	73.9	349.4
NBD1	V562I	1684G>A	372.6	588.0	1668.2	1057.0	2646.5	857.1	2398.1
NBD1	Y563N	1687T>A	45.4	52.7	224.0	193.2	363.7	60.0	393.1
NBD1	Y563D	1687T>G	45.8	50.0	210.6	192.9	388.1	61.2	405.0
NBD1	Y569D	1705T>G	48.0	54.2	232.4	205.6	408.2	68.7	317.7
NBD1	P574H	1721C>A	53.0	60.5	231.5	202.9	440.3	66.5	359.9
NBD1	F575Y	1724T>A	82.8	86.6	238.0	227.5	566.8	65.9	502.7
NBD1	G576A	1727G>C	432.4	731.3	2367.8	1400.0	3352.2	1119.8	2827.2
NBD1	D579G	1736A>G	183.5	187.6	406.5	397.5	1000.0	265.0	1062.2
NBD1	E588V	1763A>T	190.4	206.6	452.9	417.2	1008.8	306.4	1234.4
NBD1	S589T	1766G>C	398.2	672.7	1977.4	1170.4	2831.8	1017.2	3066.6
NBD1	I601F	1801A>T	84.0	88.7	249.3	236.1	549.8	83.2	547.5
NBD1	H609R	1826A>G	48.1	53.3	223.2	201.4	421.3	61.9	321.8
NBD1	A613T	1837G>A	53.9	58.7	226.7	208.1	413.0	69.6	487.2
NBD1	D614G	1841A>G	58.6	64.8	233.3	211.1	448.3	77.0	396.7
NBD1	I618T	1853T>C	60.2	67.1	232.6	208.8	470.6	51.8	416.3
NBD1	G622D	1865G>A	88.2	111.2	292.8	232.4	905.6	89.7	1056.1
NBD1	G628R	1882G>A	54.6	60.4	224.4	202.9	418.9	65.9	361.3
NBD1	G628R	1882G>C	57.6	67.0	228.1	196.3	397.8	75.0	403.5

R-Domain	R668C	2002C>T	385.7	687.5	2157.4	1210.5	2900.0	983.3	2654.3
R-Domain	S737F	2210C>T	443.4	742.0	2283.8	1364.7	3272.8	1087.9	3470.8
R-Domain	P750L	2249C>T	160.3	223.9	506.8	362.9	1228.0	240.3	1280.5
R-Domain	R751L	2252G>T	411.5	686.1	1999.5	1199.2	3009.8	1051.4	3209.0
R-Domain	V754M	2260G>A	433.7	725.5	2201.5	1316.1	3189.0	1073.5	2955.2
R-Domain	I807M	2374C>G	436.8	733.4	2231.6	1329.2	3250.5	1115.9	3462.4
R-Domain	E822K	2464G>A	249.3	491.1	1253.1	636.2	2184.0	533.8	2613.0
R-Domain	D836Y	2506G>T	370.7	583.9	1654.2	1050.2	2714.7	880.0	2940.0
TMD2	T854T	2562T>A	433.3	733.5	2190.8	1294.4	3212.5	1088.0	2943.4
TMD2	T854T	2562T>C	425.3	718.9	2246.9	1329.4	3270.3	1063.5	3026.5
TMD2	T854T	2562T>G	423.3	724.3	2202.3	1287.0	3164.3	984.6	2855.7
TMD2	S912X	2735C>A	47.6	56.3	239.3	202.0	386.5	59.3	333.5
TMD2	S912L	2735C>T	322.3	499.0	1453.3	938.8	2355.8	772.4	2700.5
TMD2	D924N	2770G>A	434.4	830.3	2508.3	1312.3	3630.0	1167.4	3778.7
TMD2	L927P	2780T>C	244.7	320.6	884.2	674.9	1760.4	448.9	1961.3
TMD2	R933G	2797A>G	508.9	913.5	2901.7	1616.6	3938.5	1378.4	3901.7
TMD2	H939R	2816A>G	407.4	782.1	2466.8	1285.0	3494.1	1092.8	3314.0
TMD2	S945L	2834C>T	123.2	191.2	440.3	283.7	1162.9	145.6	1073.7
TMD2	M952T	2855T>C	415.2	708.6	2118.3	1241.2	3115.0	1066.4	3444.4
TMD2	M952I	2856G>A	340.7	628.8	1690.3	915.9	2902.7	766.8	3072.3
TMD2	Q966=	2988G>A	425.2	720.3	2243.3	1324.2	3196.2	1090.7	2969.5
TMD2	L967S	2900T>C	283.5	369.3	948.6	728.2	1851.9	564.9	1950.3
TMD2	G970R	2908G>C	449.5	727.3	2197.9	1358.5	3224.6	1163.3	2968.1
TMD2	G970D	2909G>A	385.6	618.5	1860.7	1159.9	2738.5	983.2	2637.1
TMD2	S977F	2930C>T	393.5	711.8	2203.6	1218.2	3276.5	922.4	2992.2
TMD2	I980K	2939T>A	72.3	98.3	305.1	224.4	667.3	64.3	653.7
TMD2	L997F	2991G>C	426.1	745.3	2268.4	1297.0	3451.1	1034.7	3177.2
TMD2	Y1014C	3041A>G	383.5	692.4	2056.6	1139.1	3082.9	980.2	3301.6
TMD2	F1016S	3047T>C	168.1	297.3	638.2	360.9	1677.5	279.8	2221.3
TMD2	I1027T	3080T>C	354.9	643.9	1869.2	1030.2	2539.9	821.7	2347.2
TMD2	Y1032C	3095A>G	82.0	111.6	319.0	234.5	1944.2	107.4	795.7
TMD2	T1036N	3107C>A	60.9	101.2	355.1	213.9	1135.0	72.8	600.0
TMD2	F1052V	3154T>G	395.0	773.5	2444.4	1248.4	3204.5	1056.4	2959.3
TMD2	T1053I	3158C>T	414.0	728.8	2292.3	1302.3	3074.0	1050.1	3094.2
TMD2	H1054D	3160C>G	60.4	94.7	324.2	206.7	1086.2	69.2	715.7
TMD2	K1060T	3179A>C	383.2	614.0	1808.3	1128.6	2782.9	927.8	3104.3
TMD2	G1061R	3181G>C	52.6	65.0	249.2	201.8	546.2	62.1	365.4
TMD2	R1066C	3196C>T	45.7	48.3	205.0	194.1	363.4	84.6	340.9
TMD2	R1066H	3197G>A	56.4	81.3	298.1	207.0	834.2	68.0	549.4
TMD2	A1067T	3199G>A	281.5	493.7	1310.8	747.5	2405.3	636.4	3089.7
TMD2	G1069R	3205G>A	431.1	711.9	2196.8	1330.2	3182.2	1023.8	2888.1
TMD2	R1070W	3208C>T	185.2	284.8	662.3	430.7	1656.2	276.8	1986.6
TMD2	R1070Q	3209G>A	455.0	770.7	2375.7	1402.4	3362.0	1079.4	2995.7
TMD2	F1074L	3222T>G	171.5	366.4	792.9	371.2	1998.0	256.1	2094.1
TMD2	L1077P	3230T>C	53.9	63.8	243.3	205.5	556.9	69.2	415.3
TMD2	H1085P	3254A>C	49.1	51.7	212.5	201.6	422.9	52.1	352.6

TMD2	H1085R	3254A>G	63.6	86.7	291.6	214.0	803.4	63.1	701.9
TMD2	W1089X	3266G>A	48.8	57.2	241.3	205.9	395.7	70.2	347.5
TMD2	Y1092X	3276C>A	50.1	59.3	248.8	210.1	401.6	79.4	340.6
TMD2	Y1092X	3276C>G	49.4	58.3	248.4	210.3	417.7	67.5	323.0
TMD2	W1098R	3292T>C	54.0	63.3	246.3	210.2	404.0	63.8	307.5
TMD2	W1098C	3294G>C	55.4	76.0	287.1	209.3	817.8	61.1	624.1
TMD2	W1098C	3294G>T	64.0	94.7	327.2	221.3	818.4	56.4	572.6
TMD2	F1099L	3297C>A	113.3	305.3	776.0	288.0	2032.1	136.4	2158.9
TMD2	M1101K	3302T>A	51.9	62.4	242.6	201.6	405.8	62.5	368.7
TMD2	M1101R	3302T>G	47.0	54.3	257.7	223.1	412.4	67.8	365.0
TMD2	E1104X	3310G>T	47.5	53.6	219.4	194.7	389.8	59.6	318.5
TMD2	S1118F	3353C>T	214.9	371.1	860.8	498.6	2014.8	357.4	2446.1
TMD2	I1139V	3415A>G	411.6	701.1	2081.4	1221.8	3022.8	1045.1	3351.8
TMD2	D1152H	3454G>C	415.2	694.0	1966.9	1176.8	2860.6	1016.0	2914.4
TMD2	V1153E	3458T>A	148.4	194.8	396.8	302.3	979.7	148.5	972.9
TMD2	L1156=	3466T>C	418.4	781.0	2424.3	1299.0	3166.7	1011.0	2753.5
TMD2	L1156=	3468G>A	452.7	748.5	2163.2	1308.2	3213.4	1114.5	2955.7
TMD2	L1156F	3468G>T	429.7	765.3	2222.8	1248.2	3263.2	1046.3	3034.5
TMD2	L1156=	3468G>A	416.1	695.5	2192.4	1311.9	3186.0	1050.1	3414.1
TMD2	R1158X	3472C>T	56.5	72.4	281.5	219.8	447.2	79.9	345.4
TMD2	S1159F	3476C>T	305.9	551.5	1424.1	789.8	2380.9	689.4	2563.5
TMD2	S1159P	3475T>C	393.1	761.1	2114.6	1092.2	3265.2	930.5	3404.4
NBD2	R1162X	3484C>T	51.0	56.9	230.0	206.1	402.3	80.3	308.1
NBD2	R1162L	3485G>T	309.0	496.2	1330.1	828.4	2337.6	705.6	2391.8
NBD2	S1196X	3587C>G	318.2	437.3	1322.6	962.4	2054.8	764.6	1819.5
NBD2	W1204X	3611G>A	327.5	413.6	1226.5	971.1	1896.7	838.2	1871.5
NBD2	W1204X	3612G>A	294.3	338.0	1052.3	916.3	1736.5	654.1	1301.8
NBD2	I1234V	3700A>G	420.4	727.9	2304.5	1331.1	3352.8	1119.6	3264.0
NBD2	S1235R	3705T>G	427.2	773.8	2436.0	1344.9	3377.5	1069.0	3248.1
NBD2	V1240G	3719T>G	259.0	561.6	1454.7	671.0	1793.5	759.4	2301.6
NBD2	G1244E	3731G>A	445.1	751.4	2281.9	1351.9	3353.1	1078.5	3027.0
NBD2	T1246I	3737C>T	439.7	741.3	2179.2	1292.6	3285.8	1041.2	2973.8
NBD2	G1249R	3745G>A	414.4	730.8	2150.7	1219.5	3086.7	1049.5	3486.7
NBD2	S1251N	3752G>A	424.3	833.9	2660.0	1353.3	3312.6	1103.4	2909.7
NBD2	S1255P	3763T>C	422.0	712.3	2123.7	1258.2	3161.5	1066.1	3152.1
NBD2	I1269N	3806T>A	225.1	325.3	800.2	553.7	1418.7	506.9	1529.0
NBD2	D1270N	3808G>A	412.4	699.6	2106.7	1241.8	3151.4	998.4	2873.2
NBD2	W1282X	3846G>A	114.0	150.2	464.8	352.7	899.7	174.9	712.3
NBD2	W1282R	3844T>C	145.0	170.8	428.1	363.3	949.0	225.5	916.7
NBD2	R1283M	3848G>T	188.2	234.2	525.8	422.4	1150.6	362.9	1436.7
NBD2	R1283S	3849G>C	192.0	255.2	617.6	464.6	1219.6	343.1	1476.5
NBD2	Q1291H	3873G>C	438.7	735.9	2147.7	1280.1	3235.2	1075.4	2985.2
NBD2	Q1291R	3872A>G	438.0	777.1	2464.0	1388.8	3453.4	1154.4	3497.3
NBD2	V1293G	3878T>G	431.3	720.1	2223.0	1331.4	3314.1	1118.6	3486.8
NBD2	N1303K	3909C>G	88.4	91.7	271.8	261.8	609.5	118.4	506.6
NBD2	Q1313X	3937C>T	50.3	57.5	231.0	202.2	403.6	72.6	335.0

NBD2	L1324P	3971T>C	80.7	78.4	241.7	248.6	548.5	106.9	536.9
NBD2	L1335P	4004T>C	84.5	79.9	229.6	242.9	563.9	100.9	446.9
NBD2	G1349D	4046G>A	416.5	682.8	2008.6	1225.3	3062.5	1013.7	2839.5
NBD2	I1366N	4097T>A	168.8	206.7	499.7	408.1	1048.4	249.7	993.5
NBD2	H1375P	4124A>C	405.3	657.0	2030.5	1252.7	3171.4	968.5	3070.1
NBD2	L1480P	4439T>C	383.9	621.1	1747.4	1080.3	2658.3	964.3	3053.1
